# Innovative Left Ventricular Assist Device in High-fidelity Patient Simulator

**DOI:** 10.7759/cureus.7763

**Published:** 2020-04-21

**Authors:** Wayne Lindsay, Tiffany Nelms, Sean O'Hara, Zachary Sletten

**Affiliations:** 1 Emergency Medicine, Brooke Army Medical Center, San Antonio, USA; 2 Simulation, Brooke Army Medical Center, San Antonio, USA; 3 Emergency Medicine, San Antonio Military Medical Center, San Antonio, USA

**Keywords:** lvad, left ventricular assist device, simulator, simulation, emergency medicine, lvad emergency, emergency room

## Abstract

Left ventricular assist devices (LVADs) are implantable mechanical devices that pump blood from the apex of the left ventricle to the aorta in order to assist the forward flow of blood; they are most commonly used as a bridge to transplant for patients with heart failure. As of February 2019, a total of 25,145 patients with ventricular assist devices have been reported in the Interagency Registry for Mechanically Assisted Circulatory Support (Intermacs). As this number continues to grow, more and more of these patients will inevitably be seen in the acute care setting outside of their defined LVAD center. Currently, however, LVAD emergencies represent a high-acuity low-occurrence event with limited opportunities for exposure and mastery for most physicians. Therefore, a growing need exists for emergency care providers to familiarize themselves with these devices and the management of LVAD emergencies. We present a novel model for the simulation of LVAD emergencies created through simple modifications of a Laerdal 3G Manikin.

## Introduction

The left ventricular assist device (LVAD) was initially developed in the 1960s as a bridge to cardiac transplant. Since the number of patients awaiting cardiac transplant has continued to rise, LVADs have also commonly become destination therapy. This device may also be used for temporary support in cardiomyopathies and patients diagnosed with New York Association’s class IV heart failure [1]. As of 2019, there were an estimated 25,145 patients with LVAD devices and the number continues to grow [1]. These patients are primarily managed at centers with both LVAD-trained cardiologists and support staff. Yet, with the increasing number of patients relying on these devices, it is inevitable that acute care will need to be provided outside of dedicated LVAD centers. A recent study suggests that the average patient with an LVAD will be seen in the emergency department (ED) seven times a year, with a 64% admission rate [2]. Patients with an LVAD are at high risk of experiencing a complication, and, unfortunately, there are a variety of emergent complications associated with having an LVAD, including bleeding, thrombosis, and infection [3]. Additionally, there are many components of the physical examination unique to patients with an LVAD, including blood pressure measurement, cardiac auscultation, and assessment of the device and its components [3]. As such, it is imperative that emergency providers gain familiarity with the device and the notable physiological changes in these patients. For an emergency physician not routinely caring for patients with an LVAD, LVAD emergencies represent a high-acuity low-occurrence (HALO) event with limited opportunities for training and familiarization.

The LVAD is composed of several key components that this simulator aims to mimic: the pump, which sits internally and pumps blood from the left ventricle into the thoracic aorta, the driveline, which is the percutaneous cable that exits the abdominal wall to connect the pump to the external components, an external computer controller, which both monitors and controls pump performance with a display screen, and a power supply, which typically rests along the patient’s hip with a backup battery located in an additional pouch [4].

The LVAD pump provides continuous forward flow from the left ventricle to the aorta; as such there are notable physiological changes in these patients. There is no longer a definable systolic and diastolic component to the cardiac cycle. These patients will lack a palpable pulse, and healthcare providers will not be able to record a traditional blood pressure [5]. This low-cost simulator may be used to introduce these physiological changes and provide training on common LVAD pathologies such as driveline site infections, bleeding events, and power source failure.

## Technical report

This simulation was built with a Laerdal 3G Manikin (Laerdal, Stavanger, Norway); however, other manikin models may be used in its place. The following steps and figures serve to outline the simple process of configuring an LVAD simulator.

**Figure 1:**
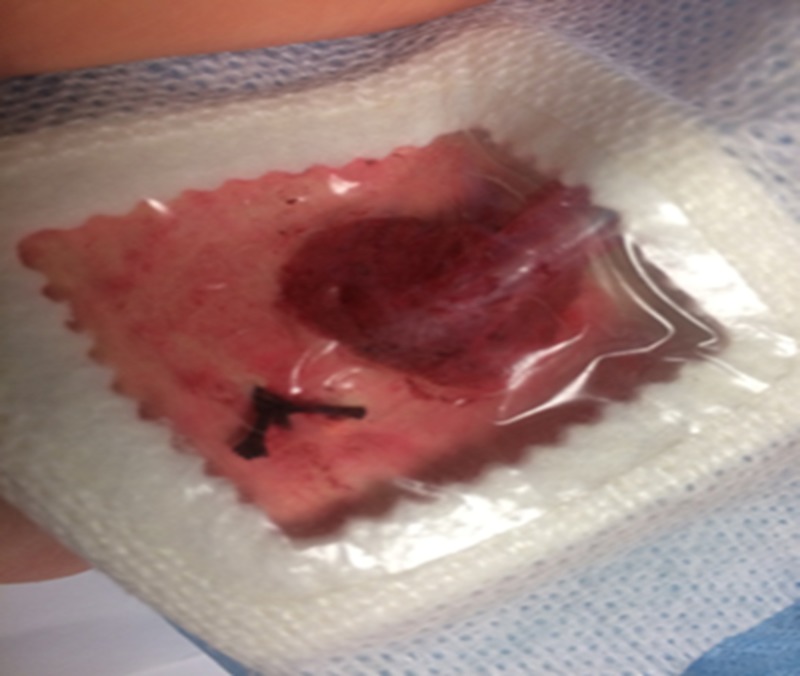
The Sorbaview 2000 window dressing

A small square of manikin-colored material should be cut approximate to the size of your intended window dressing; for this model, a Sorbaview 2000 window dressing (Centurion, Williamston, MI, USA) was used with a window of approximately 4 x 4 cm. Red grease paint and modeling clay were used for creating a moulage, representing erythema with mild bleeding as seen in Figure 1. The moulage can differ depending on the intended simulation case.

**Figure 2:**
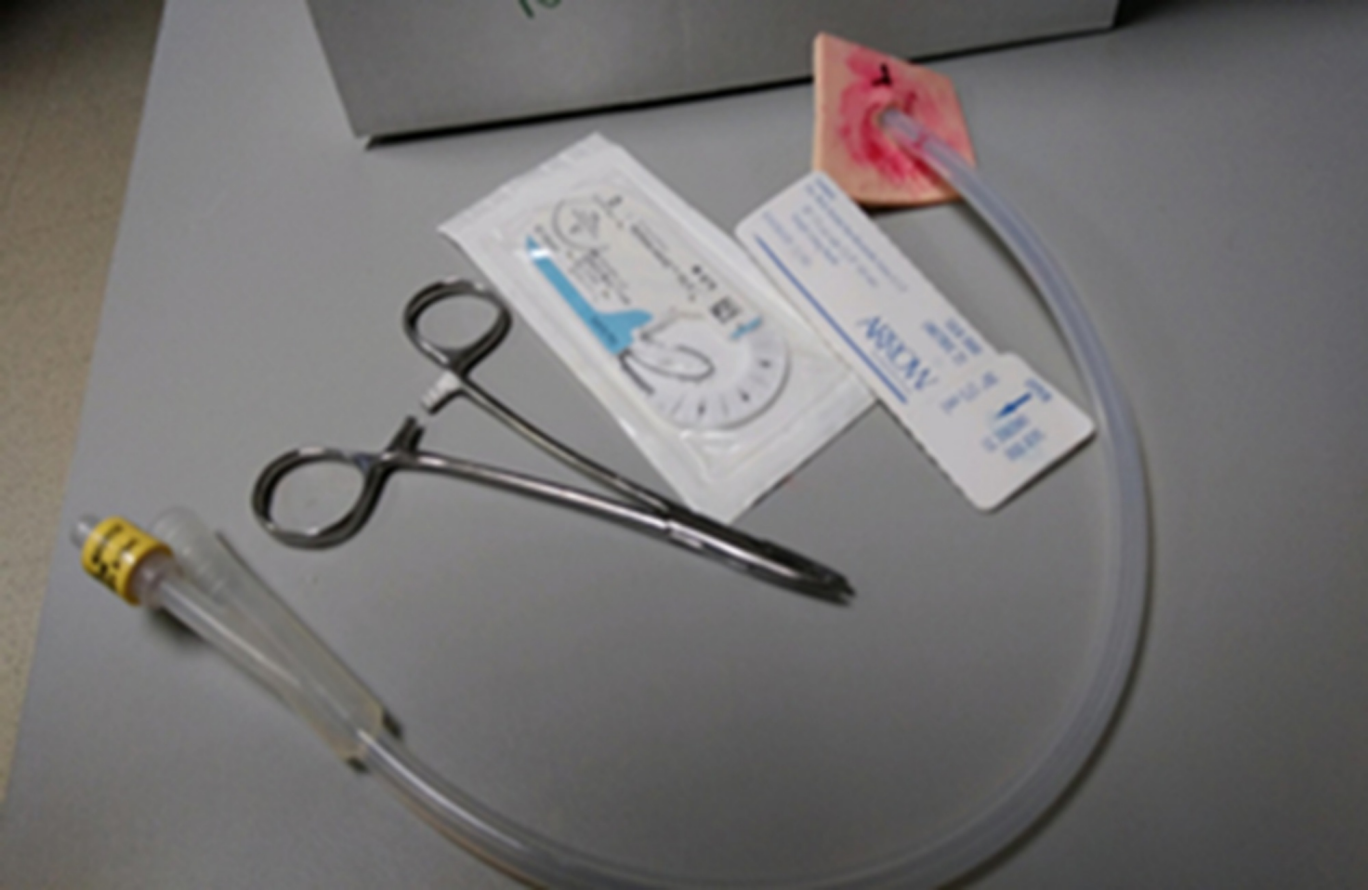
Foley catheter attached to the manikin-like material with suture and hemostat

A Bardex 20-French silicone Foley catheter (Bard Medical, Covington, GA, USA) was used to represent the driveline coming from the patient’s abdomen and connecting to the controller. The Foley catheter (Figure 2) with the tip removed was then inserted through a small cut in the dressing and sutured in place with black Ethicon braided suture on the manikin material along the edge of the window (Figure 1).

**Figure 3:**
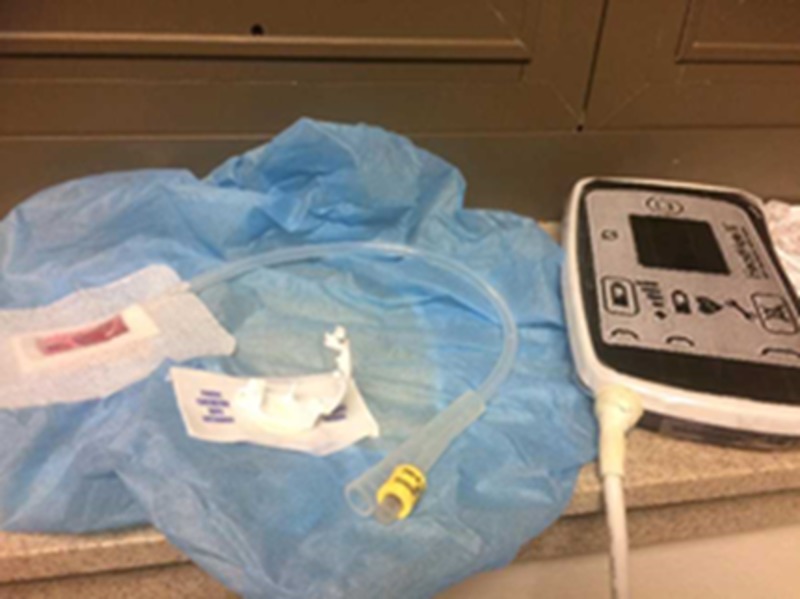
The printed controller panel attached to the hospital bed remote

A discarded PositionPro hospital bed remote (Stryker, Kalamazoo, MI, USA) was then used as the patient controller with a printed image modeled after a Heartmate III controller (Thoratec Corporation, Pleasanton, CA, USA) (Figure 3). The Foley catheter was then taped to the bed remote representing the driveline attaching to the controller, while the remote’s cord was run to a simulated battery pack.

**Figure 4:**
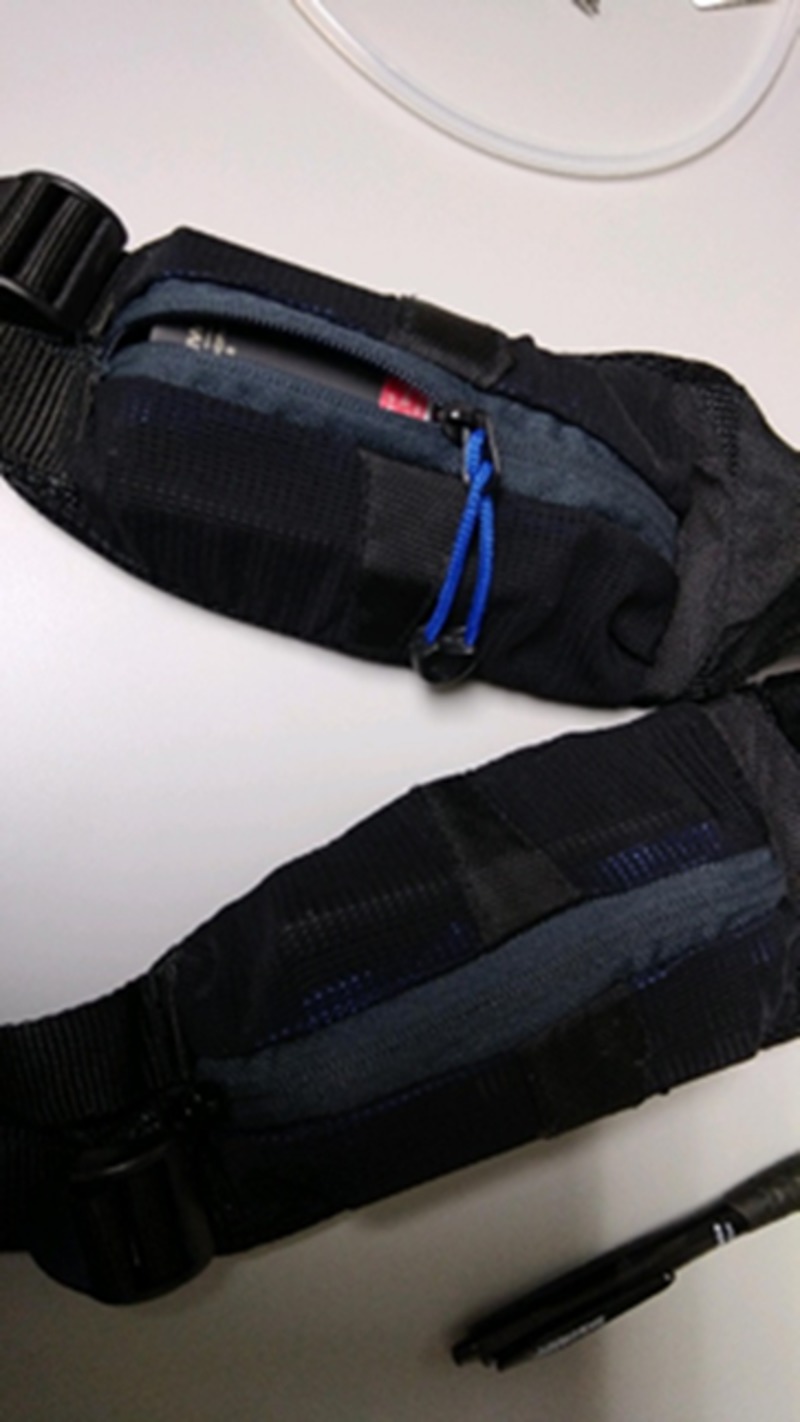
The simulated battery pack with a spare pack

**Figure 5:**
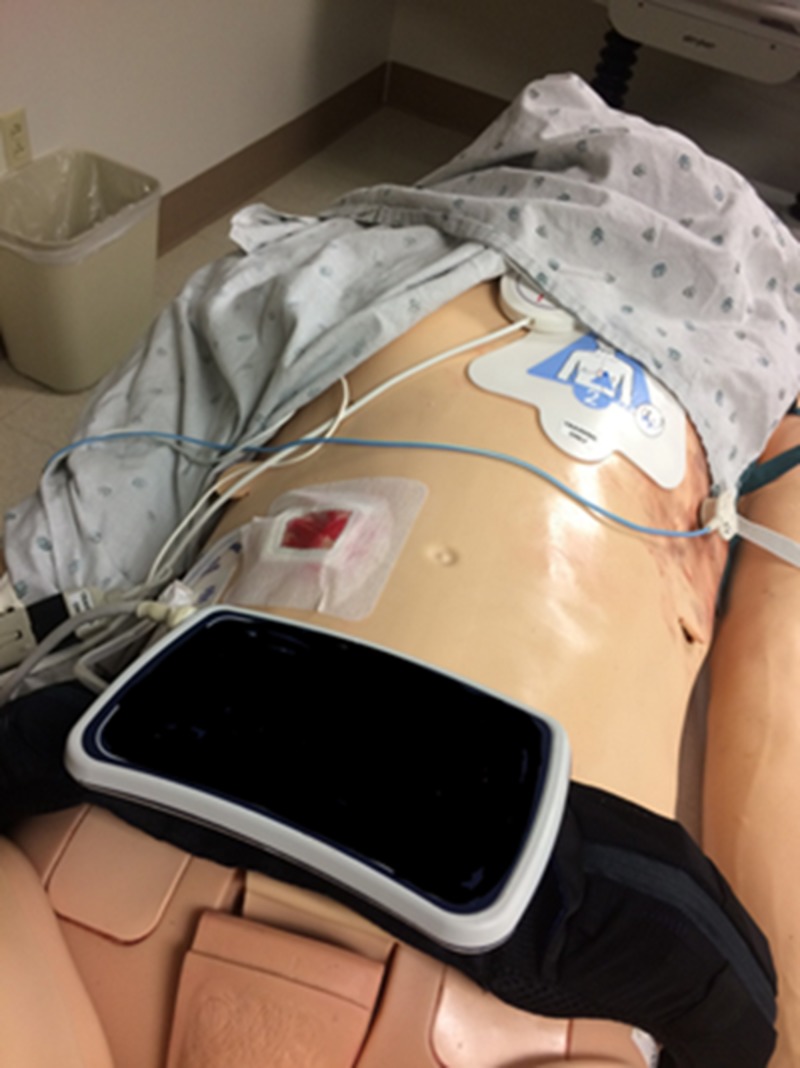
The controller resting in the middle of the battery packs

Two large squared batteries, taken from an Audio-Technica wireless microphone receiver (Audio-Technica Corp., Tokyo, Japan), were then placed in the waist packs from a hiking backpack (Figure 4) to represent the primary battery and the backup most patients will carry at all times. A fanny pack with dual pockets may also serve as the battery containers. The LVAD controller was then connected to the front of the waist packs between the batteries (Figure 5).

**Figure 6:**
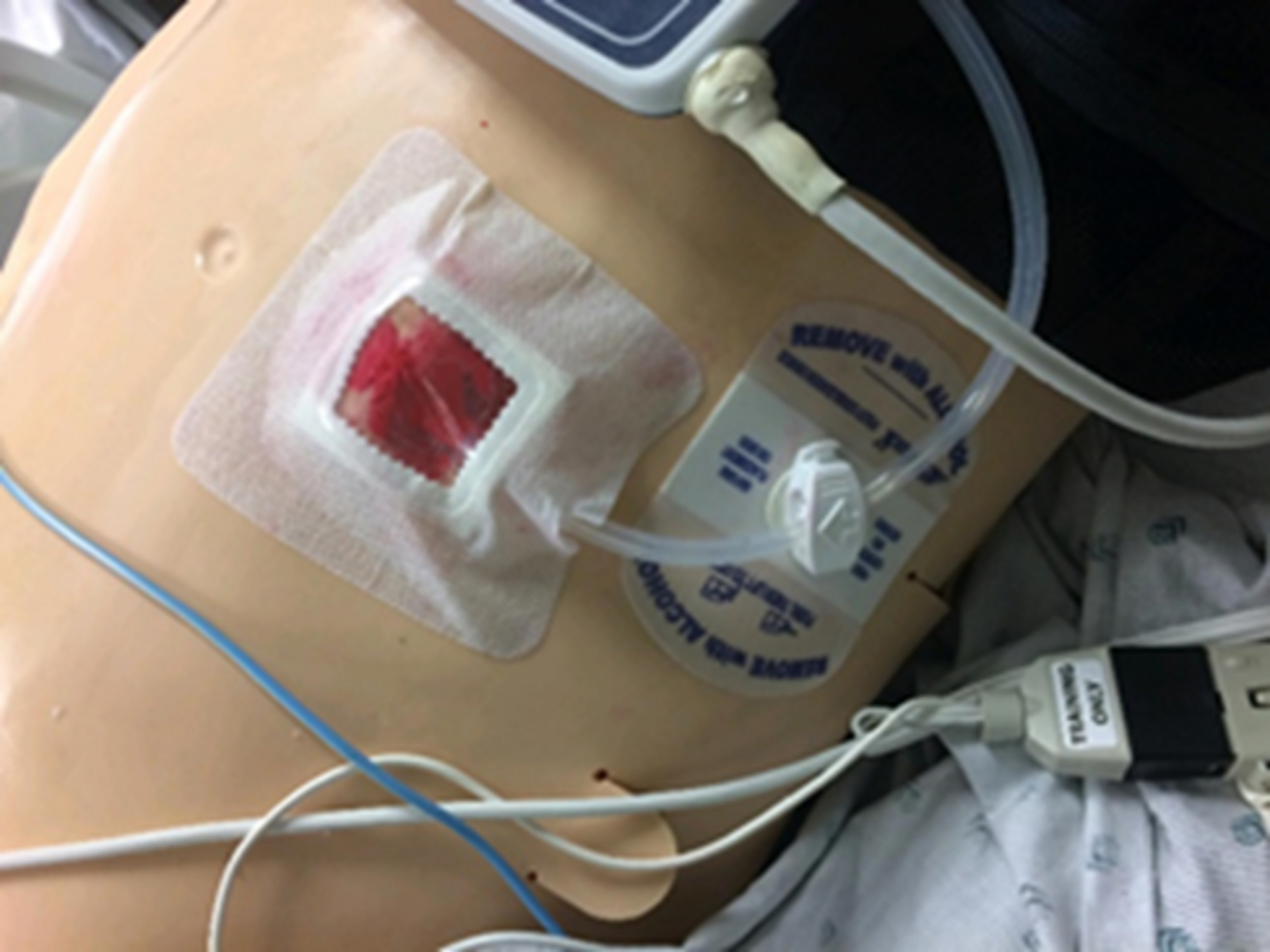
The cloth tape dressing in place on the right abdomen

The Sorbaview 2000 window dressing piece was then adhered to the right mid-abdomen with the Foley catheter portion feeding out laterally connecting to the simulated controller (Figure 6). The driveline may exit a patient from either their right or left abdomen to connect to the controller [4].

**Figure 7:**
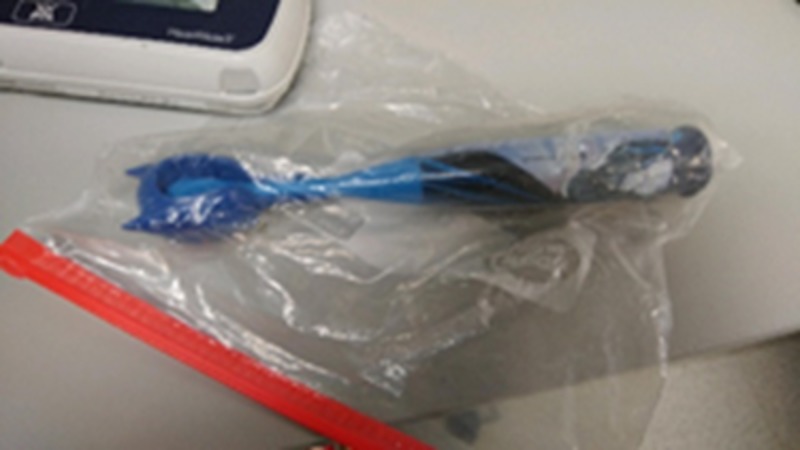
The mechanical toothbrush used to represent the pump

Finally, an electric toothbrush was placed in the manikin’s chest cavity and turned on to represent the LVAD pump functioning within the heart, creating the continuous hum that would be heard when auscultating these patients (Figure 7).

## Discussion

The number of patients with LVADs will continue to grow and will inevitably come under the care of healthcare providers unfamiliar with LVADs. Patients with LVADs have frequent ED visits and subsequent admissions with a wide array of emergent complications [2]. These encounters represent HALO events for the majority of emergency physicians. There are many aspects of managing LVAD patients that are unique and potentially unfamiliar to include techniques for measuring blood pressure and troubleshooting the device components [3]. LVAD simulation training provides life-saving education and guidance on how to care for these patients.

With only minor modifications, simulation centers can create a functioning LVAD simulator that can be incorporated into their simulation curriculum. The Brooke Army Medical Center (BAMC) Simulation Department has successfully incorporated this simulator into training for the BAMC Emergency Medicine Residency with cases including ventricular fibrillation, driveline bleeding, and surgical site infection.

## Conclusions

The basic modifications outlined in this article allow for the creation of a high-fidelity low-cost LVAD simulator that can be used to provide familiarity with LVAD physiology and education on the complex pathology unique to these patients. The model is both reusable and can be reverted back to the manikin’s original state with relative ease.
